# Gene expression profiling identifies potential biomarkers for vaso-occlusive episodes in sickle cell disease

**DOI:** 10.1172/jci.insight.193359

**Published:** 2026-03-09

**Authors:** Varsha Bhat, Justin J. Yoo, Srija Ponna, Alka A. Potdar, Ashwin P. Patel, G. Karen Yu, Greg Gibson, Vivien A. Sheehan

**Affiliations:** 1 Center for Integrative Genomics, School of Biological Sciences, Georgia Institute of Technology, Atlanta, Georgia, USA.; 2 Aflac Cancer and Blood Disorders Center, Children’s Healthcare of Atlanta, Emory University School of Medicine, Atlanta, Georgia, USA.; 3 Pfizer, South San Francisco, California, USA.

**Keywords:** Hematology, Inflammation, Biomarkers, Expression profiling, Pain

## Abstract

Vaso-occlusive episodes (VOEs) or acute pain events, involving complex interactions between sickle erythrocytes and other blood cells, are a hallmark of sickle cell disease (SCD). In this study, we analyzed changes in peripheral blood transcriptomes between steady state and VOEs in individuals with SCD. We followed a cohort of 174 individuals with SCD with or without chronic pain and collected peripheral blood at clinic visits (steady state) and during hospitalizations (VOEs). We performed RNA-Seq profiling of CD45^+^ leukocytes and CD71^+^ erythroid cells. Pathways linked to complement activation, coagulation, and IL-6/JAK/STAT3 signaling were enriched during VOEs in the CD45^+^ cells. Contrastingly, the CD71^+^ cells showed an enrichment of pathways related to the cell cycle, such as mTORC1 signaling and the G_2_M checkpoint during VOEs. We then analyzed the expression changes of genes in patients with longitudinal data to determine potential biomarkers for VOEs. Expression of 4 genes — *FAM20A*, *IL1B*, *MS4A4A*, and *SERPINB2* — was elevated during VOEs compared with steady state in the majority of patients. Furthermore, our results indicate that patients experiencing chronic pain exhibited 44% increased enrichment of significant pathways during VOEs when compared with patients without chronic pain.

## Abstract 2

RNA-Seq profiling of immune and erythroid cells from SCD patients shows multiple genes and biological pathways altered during vaso-occlusive episodes in sickle cell disease

## Graphical abstract

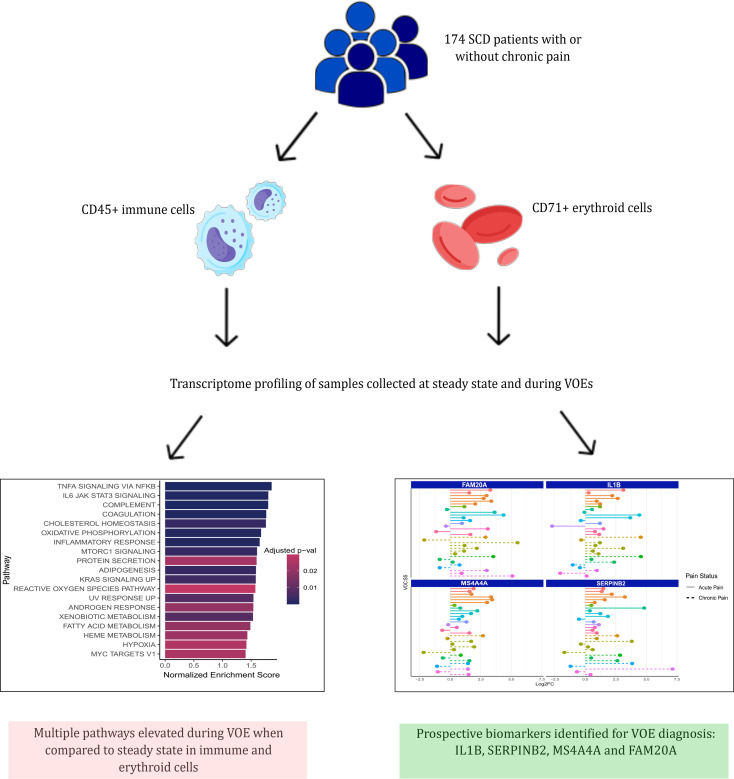

## Introduction

Sickle cell disease (SCD) is a genetic disorder caused by a point mutation in the β-globin gene, replacing the hydrophilic glutamic acid with hydrophobic valine (E6V). SCD affects more than 10 million people worldwide, with the highest disease prevalence in sub-Saharan Africa (1). Vaso-occlusive episodes (VOEs) are the primary symptom of SCD and a major cause of hospitalizations and morbidity. VOEs may be initiated by adhesive interactions between sickled erythrocytes, leukocytes, endothelial cells, and activated platelets, leading to obstruction of the microvasculature. Obstruction causes ischemia, leading to tissue damage and severe pain. Pain often occurs in the abdomen, chest, back, and extremities. A typical VOE lasts 5–7 days (2); adults with SCD generally experience VOEs requiring acute care 2–3 times a year (3). However, severity and incidence vary considerably among individuals and across β-globin genotypes. Up to half of adults with SCD and 10%–30% of pediatric patients with SCD develop chronic pain, defined as pain lasting 6 months or more, present on most days (4). SCD pain is managed acutely with opioids and nonsteroidal antiinflammatory drugs (5). Although the causes of chronic pain are multifactorial, the repeated use of opioids can trigger opioid-induced hyperalgesia (4) and is correlated with a poorer quality of life (6).

The molecular mechanisms underlying the development of chronic pain in SCD and other conditions are a matter of intense investigation. VOE is diagnosed based on patient-reported pain. However, the experience of pain is subjective, and there is high variability between patients with respect to perception of pain intensity, response to therapy, and tolerance (7). It can be difficult for patients and providers to differentiate between acute and chronic pain, or a mixed picture of acute pain overlaid on chronic pain. At present, we lack objective and quantitative biomarkers for VOEs (8); this information is critical for defining endpoints in clinical trials when testing therapies for pain and developing effective treatment approaches. The absence of biomarkers typically seen in VOEs can aid in the diagnosis of chronic pain, and facilitate the institution of opioid-sparing, multidisciplinary approaches that more effectively address the unique biology of chronic pain and the comorbidities that exacerbate chronic pain. Several potential VOE biomarkers have been proposed, but none has been validated so far, particularly in longitudinal cohorts.

In this study, we investigated the changes in the transcriptome of patients with SCD hospitalized for pain compared to their steady-state levels. We examined these changes in individuals with SCD who are pain-free between VOEs (no chronic pain) and in those who are not pain-free between admissions, with pain most days for at least 6 months (chronic pain). We performed bulk RNA-Seq on 2 cell types: CD45^+^ cells and CD71^+^ cells. While CD45^+^ cells are cells of the lymphohematopoietic lineage, including all leukocytes, the transferrin receptor CD71 is primarily expressed on erythroid precursor cells and a small subset of neutrophils (9). Since pain events are thought to mainly involve interactions between leukocytes and erythrocytes (10), we chose to analyze these 2 cell types.

## Results

### Impact of VOEs on gene expression in lymphohematopoietic cells.

We performed bulk RNA-Seq gene expression profiling of CD45^+^ cells from a dataset composed of 197 samples, with 155 samples collected at steady state and 42 samples during VOEs. Our first objective was to identify changes in gene expression in leukocytes between steady state and VOEs in patients with SCD. The mean library size for RNA-Seq was approximately 39 million reads per sample. After filtering, 28,005 genes were retained, and normalization was performed using supervised normalization of microarrays (SNM) to control for variation in cell proportions inferred from informative transcripts (see Methods).

**Figure 1:**
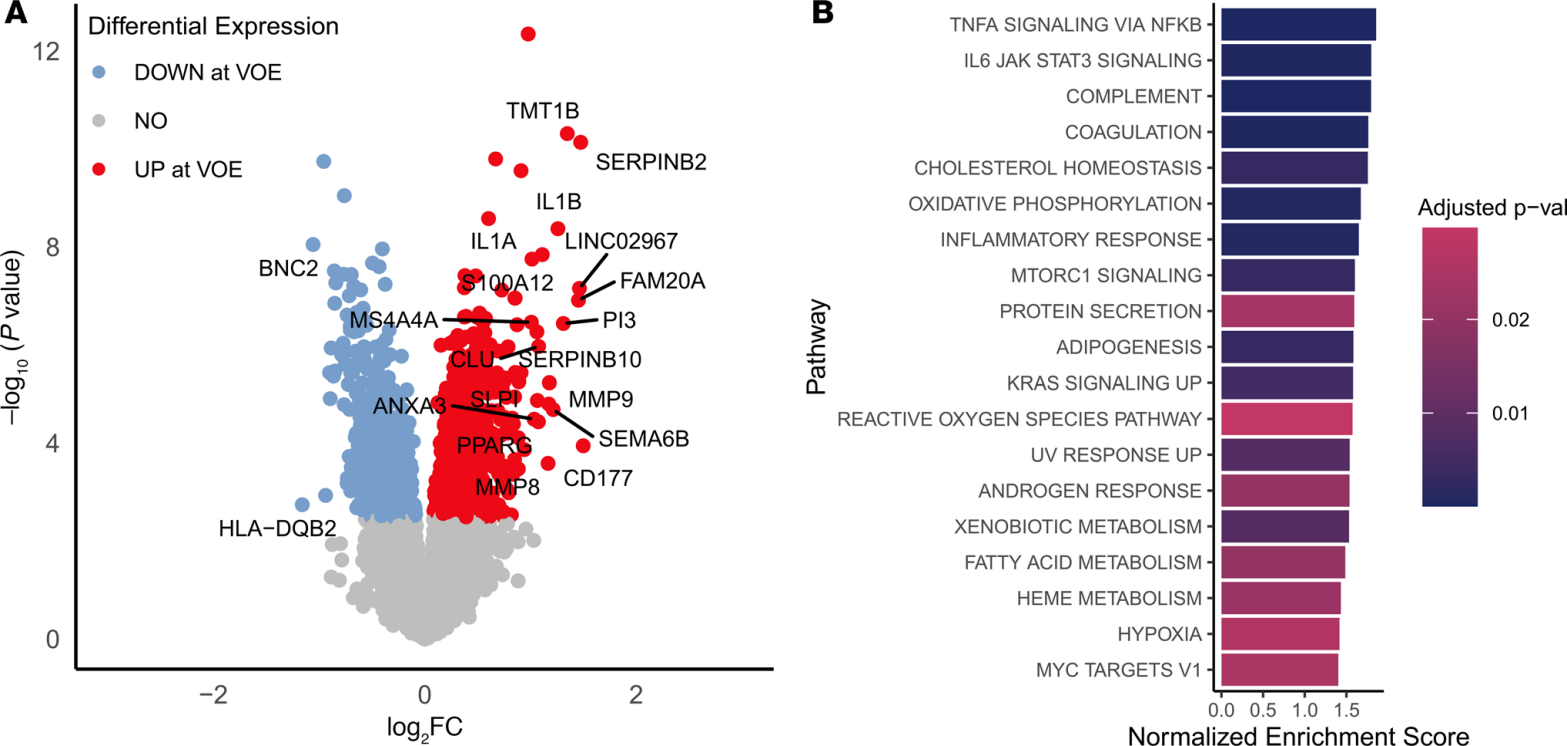
CD45^+^ cell gene expression and pathway enrichment changes in VOEs. (**A**) Volcano plot depicting differentially expressed genes in CD45^+^ cells (*n* = 197 samples). The genes in red and blue have adjusted *P* values of less than 0.05 (Benjamini-Hochberg threshold). Genes with log_2_(fold change) (log_2_FC) greater than 1.0 or less than –1.0 are labeled with gene names. (**B**) Pathway enrichment analysis results: Bar plot depicting the pathways upregulated during VOEs when compared with steady state in CD45^+^ cells of patients with no chronic pain or chronic pain (*n* = 197 samples), with colors indicating significance adjusted for multiple comparisons. None of the significant pathways were downregulated during VOEs.

We identified 1887 differentially expressed genes between steady state and VOEs in the entire cohort, with and without chronic pain, with Benjamini-Hochberg–adjusted *P* values of less than 0.05 (Supplemental Table 1; supplemental material available online with this article; https://doi.org/10.1172/jci.insight.193359DS1); 1197 genes were upregulated during VOEs (positive log_2_[fold change], red points in Figure 1A) and 690 genes were downregulated during VOEs (negative log_2_[fold change], blue points). Pathway enrichment analysis with fgsea (11) showed that numerous biological pathways associated with inflammatory response, complement activation, coagulation, and oxidative phosphorylation were elevated during VOEs. This result suggests that a vaso-occlusive pain episode is primarily associated with a biological mechanism linked to an exacerbation of inflammation (Figure 1).

**Figure 2:**
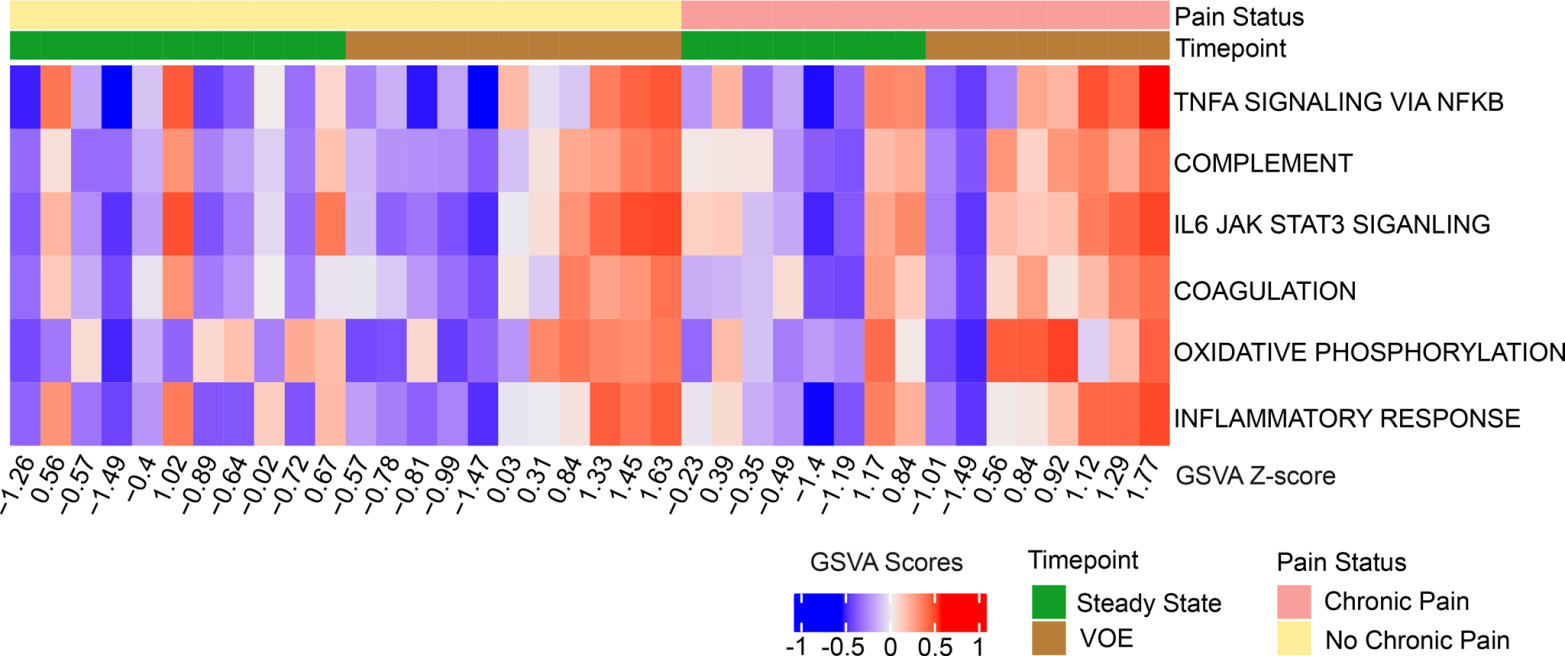
Heatmap of GSVA scores indicates changes in pathway enrichment between steady state and VOEs in patients with SCD with longitudinal data. The columns are sorted by pain status and time point. The numbers below each column indicate the *z* score for pathway enrichment in each sample, computed using the individual GSVA scores.

We further computed individualized gene set variation analysis (GSVA) scores to examine pathway enrichment at the sample level for the 19 patients with longitudinal data (samples collected at both steady state and VOEs). Figure 2 shows the GSVA scores for the top 6 pathways elevated during VOEs and with the lowest adjusted *P* values in the gene set enrichment analysis (GSEA) analysis. Although the extent of elevation of these pathways varies between samples, there is remarkable consistency in the 6 GSVA scores within each sample, further confirming the generalized inflammatory nature of the response. The enrichments do not simply reflect the abundance of T cells in the CD45^+^ population since the analysis was statistically adjusted for inferred proportions of T and B cells.

To evaluate whether the CD45^+^ cell transcriptomes of patients without chronic pain change in a similar manner during VOEs, we performed a separate analysis restricted to patients categorized as having no chronic pain in the cohort; samples were collected from 134 patients at steady state and 17 patients during VOEs. We observed 538 differentially expressed genes in patients with no chronic pain; 358 genes were upregulated during VOEs, and 180 genes were downregulated (Supplemental Table 2). All of the pathways except for IFN-α response also appeared in the analysis of all patients in the full cohort. In addition, there was significant downregulation of the allograft rejection pathway.

**Figure 3:**
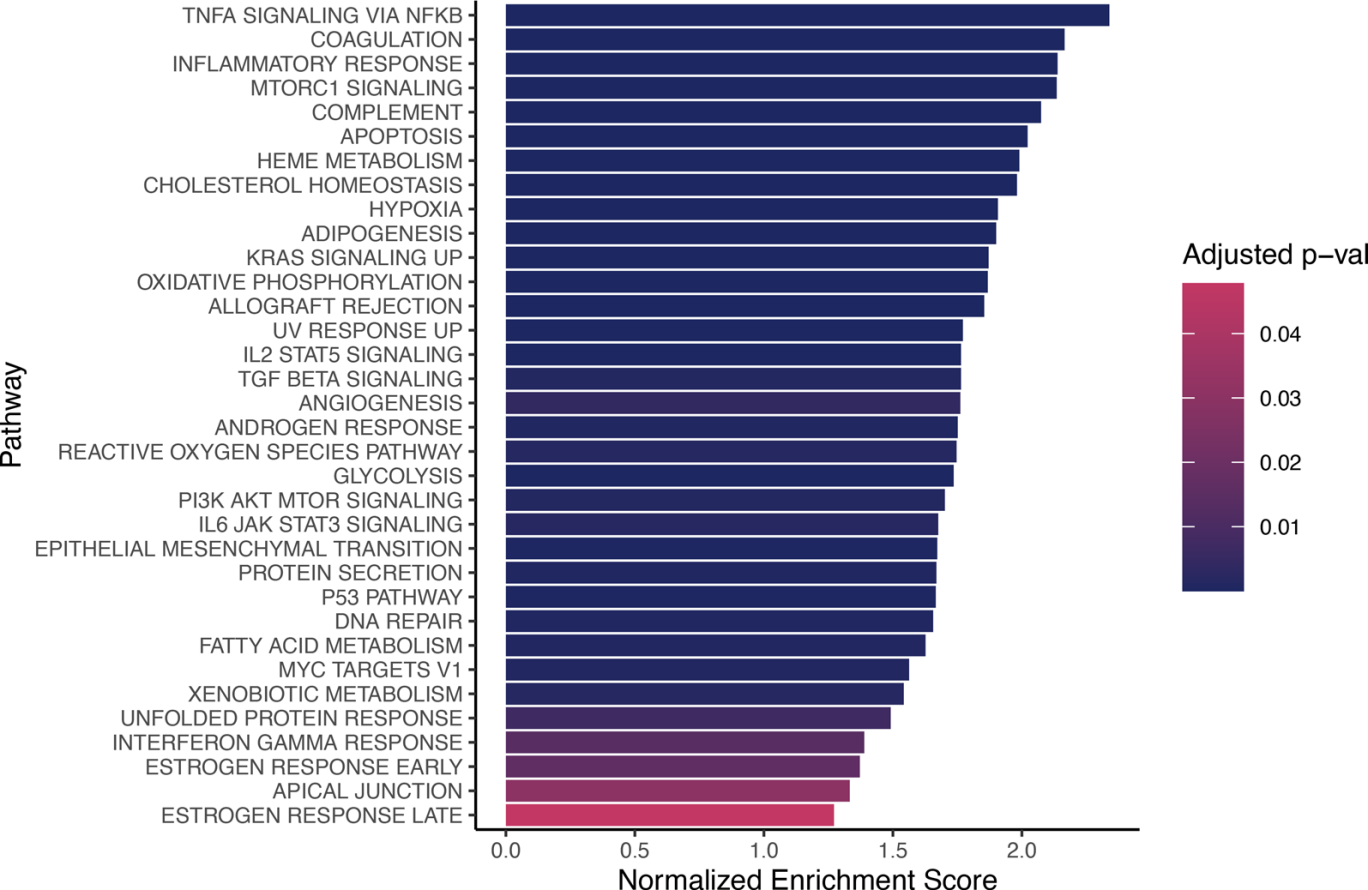
Pathway enrichment results for CD45^+^ cells from patients with chronic pain. Bar plot depicting the 34 pathways upregulated (none were downregulated) during VOEs when compared with steady state in CD45^+^ cells of patients with chronic pain (*n* = 38 samples).

Similarly, we analyzed the transcriptomic differences between steady state and VOEs in patients categorized as having chronic pain; 15 samples were collected at steady state, and 18 samples were collected during VOEs. Despite the smaller sample size, a total of 313 genes were found to be differentially expressed, with 128 genes upregulated during VOEs, and 185 downregulated during VOEs (Supplemental Table 3). Pathway enrichment analysis revealed the upregulation of 34 pathways during VOEs, with relatively higher enrichment scores compared with the GSEA results from the analysis of all patients (comparing Figure 1 and Figure 3). Almost half of the pathways upregulated during VOEs in patients with chronic pain were also elevated during VOE in the analysis of all patients. Analyses with datasets of small sample sizes are often underpowered, increasing the likelihood of detecting false positives and false negatives and potentially undermining the findings of the study (12). In order to test whether this enhanced enrichment was specific to the chronic pain set rather than an artifact of the smaller sample size, we performed a bootstrap downsampling by creating 30 random subsets of samples from patients without chronic pain and subsequently performed the same GSEA with each subset. The number of enriched pathways during VOEs when compared with steady state ranged from zero to 26, indicating that the total number of pathways observed in the chronic pain analysis is indeed higher than the number of pathways observed in the samples without chronic pain (*P* = 0.006). Similarly, the mean normalized enrichment score for the chronic pain pathways significant at *P* less than 0.01 was 1.79, which was greater than that observed in any of the 30 permutations. The mean normalized enrichment score for all pathways significant at *P* less than 0.05 in patients with chronic pain was 44% higher than the score observed for patients without chronic pain.

Due to the difference in the magnitude of enrichment between the chronic pain set and the no chronic pain set, we wanted to determine whether the transcriptome of samples collected from patients without chronic pain at steady state varied from that of chronic pain samples collected at steady state; however, we did not find any differentially expressed genes, possibly due to the small number of chronic pain samples.

### Effect of VOEs on the transcriptome of erythroid precursors.

Transcriptome profiling of CD71^+^ erythroid precursor cells was conducted on 180 samples obtained from 141 patients, with 138 samples collected at steady state and 42 samples during VOEs. The mean library size was approximately 38 million reads per sample. A total of 19,162 genes were provided as input for differential gene expression (DGE) analysis after quality control and normalization. Since CD71 is also expressed at low levels on the surface of neutrophils (9), we cannot exclude the possibility that the results may be in part due to neutrophil contamination. The gene expression levels of CD45, CD71, and CD15 (a neutrophil marker) for all CD71^+^ samples are listed in Supplemental Table 4; most samples had relatively low levels of CD15 expression compared with CD45, suggesting minimal neutrophil contamination.

**Figure 4:**
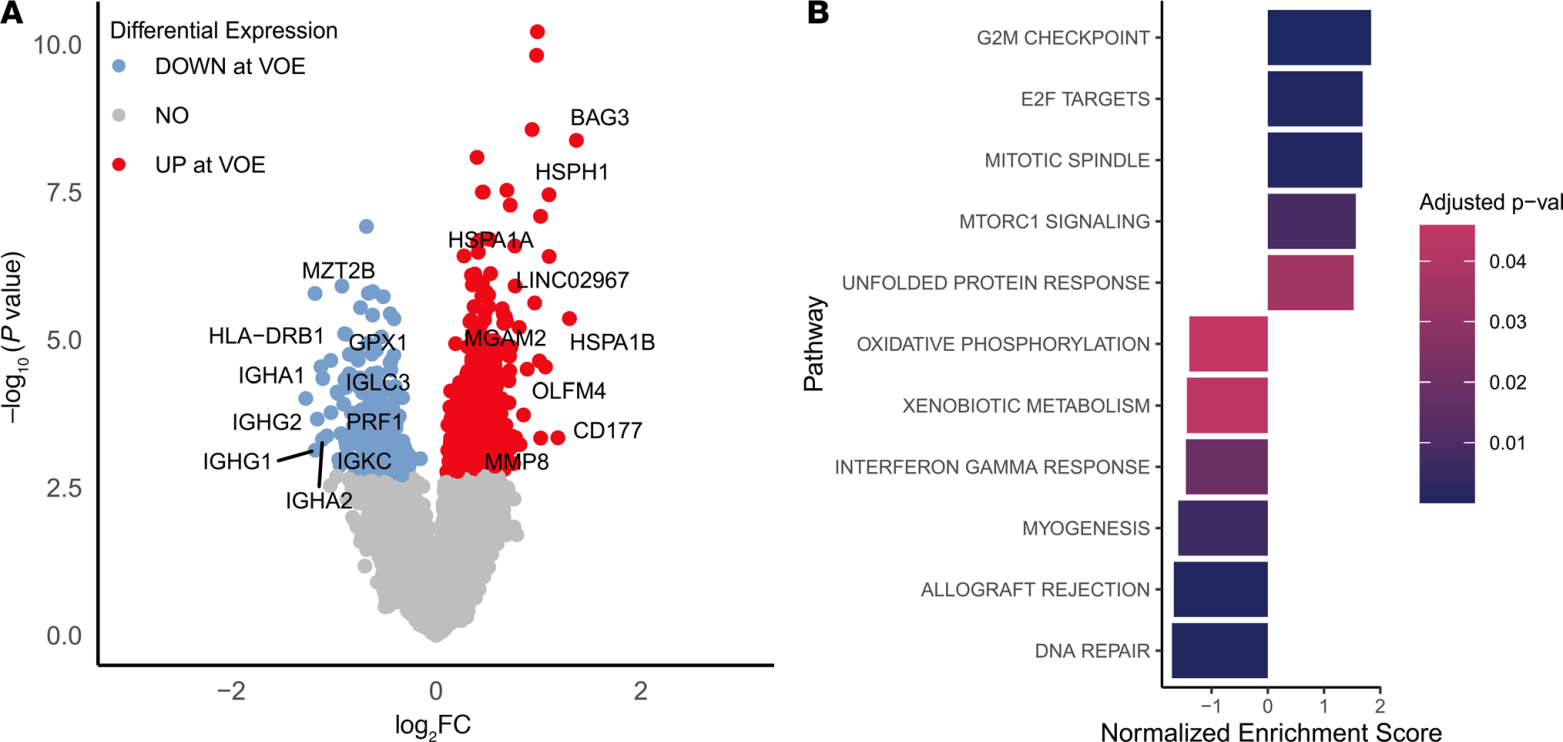
CD71^+^ cell gene expression and pathway enrichment changes in VOEs. (**A**) Volcano plot depicting genes differentially expressed between VOEs and steady state in CD71^+^ cells (*n* = 180 samples). The colored genes have adjusted *P* values of less than 0.05 (Benjamini-Hochberg threshold). Genes with log_2_(fold change) (log_2_FC) greater than 1.0 or less than –1.0 are labeled with gene names. (**B**) Pathway enrichment analysis results: Bar plot depicting the pathways upregulated or downregulated during VOEs when compared with the steady state in CD71^+^ cells of patients with no chronic pain or chronic pain (*n* = 180 samples).

DGE analysis revealed that 511 genes were upregulated and 260 genes were downregulated during VOEs in comparison with steady state (Benjamini-Hochberg–adjusted *P* < 0.05) (Supplemental Table 5). Pathways altered during VOEs were mostly linked to cell proliferation; the G_2_M checkpoint, E2F targets, and mTORC1 signaling pathways were upregulated during VOEs (Figure 4), while allograft rejection and IFN-γ signaling were downregulated.

We also analyzed the CD71^+^ cell transcriptome of patients categorized as having no chronic pain, with samples collected from 115 patients during steady state and 17 patients during VOEs. We observed 441 differentially expressed genes; 373 genes were upregulated during VOEs, and 68 genes were downregulated during VOEs (Supplemental Table 6). In addition to the pathways shown in Figure 4, the IL-6/JAK/STAT3 signaling pathway and IFN-α response pathway were elevated during VOEs, suggesting that CD71^+^ cells may be involved in immunomodulation during VOEs in patients without chronic pain; both of these pathways were also elevated during VOEs in the CD45^+^ cell transcriptome of patients without chronic pain. Future studies incorporating single-cell approaches will be useful to pinpoint the specific cell subsets that contribute to the enrichment of these pathways. We did not find any differentially expressed genes between steady state and VOEs in patients with chronic pain.

### Potential diagnostic markers for VOEs from longitudinal data.

**Figure 5:**
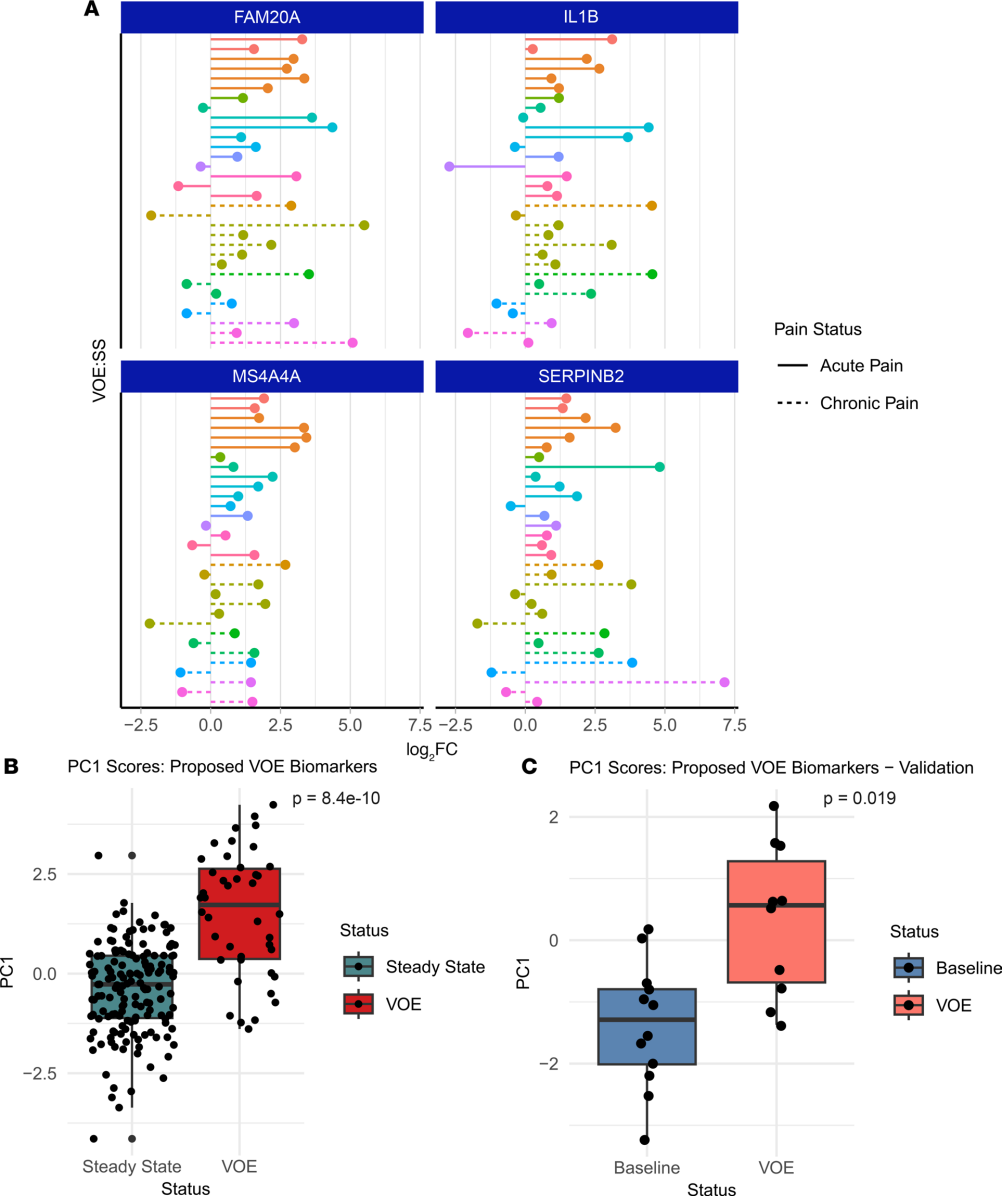
Potential VOE biomarkers. (**A**) Lollipop plots depicting the expression changes of potential diagnostic VOE biomarkers. Each point shows the log_2_(fold change) (log_2_FC) in expression of the indicated gene in each donor with paired steady-state (SS) and VOE samples. The colors denote unique patients in the dataset (*n* = 63 samples, 19 patients). Multiple VOE samples were collected for 7 patients, and multiple steady-state samples were collected for 1 patient. (**B**) Box-and-whisker plots depicting PC1 scores of the proposed biomarker genes as a function of time point (VOE/steady state) in our cohort (*n* = 197 samples). (**C**) PC1 scores of the proposed biomarker genes as a function of time point (VOE/steady state) in an external validation dataset (*n* = 22 samples) (13). Significance was determined using a 2-tailed Student’s *t* test.

To identify biomarkers for VOE diagnosis in an unbiased manner, we compared the expression of several differentially expressed genes between steady state and VOEs in the 19 SCD patients with longitudinal data, namely in this study both steady-state and VOE time points (11 without chronic pain and 8 with). We identified 4 genes that were upregulated during VOEs in more than half of the patients in the RNA-Seq data from their CD45^+^ cells and that were also associated with pathways elevated during VOEs in the analysis of the complete cohort: *FAM20A*, *SERPINB2*, *IL1B*, and *MS4A4A* (Figure 5A and Supplemental Figure 1). PC1 scores summarizing the expression of these 4 genes in the VOE group were significantly higher than in the steady-state group, both in our full dataset (*P* = 8.405 × 10^–10^) and in an external validation dataset (ref. 13) (*P* = 0.0192) (Figure 5, B and C), suggesting that these genes could be used as diagnostic biomarkers for VOEs with further independent validation. Two of these genes, *SERPINB2* and *IL1B*, also contribute heavily to the enrichment signal of multiple pathways upregulated during VOEs in the CD45^+^ cells (Supplemental Figure 2).

**Figure 6:**
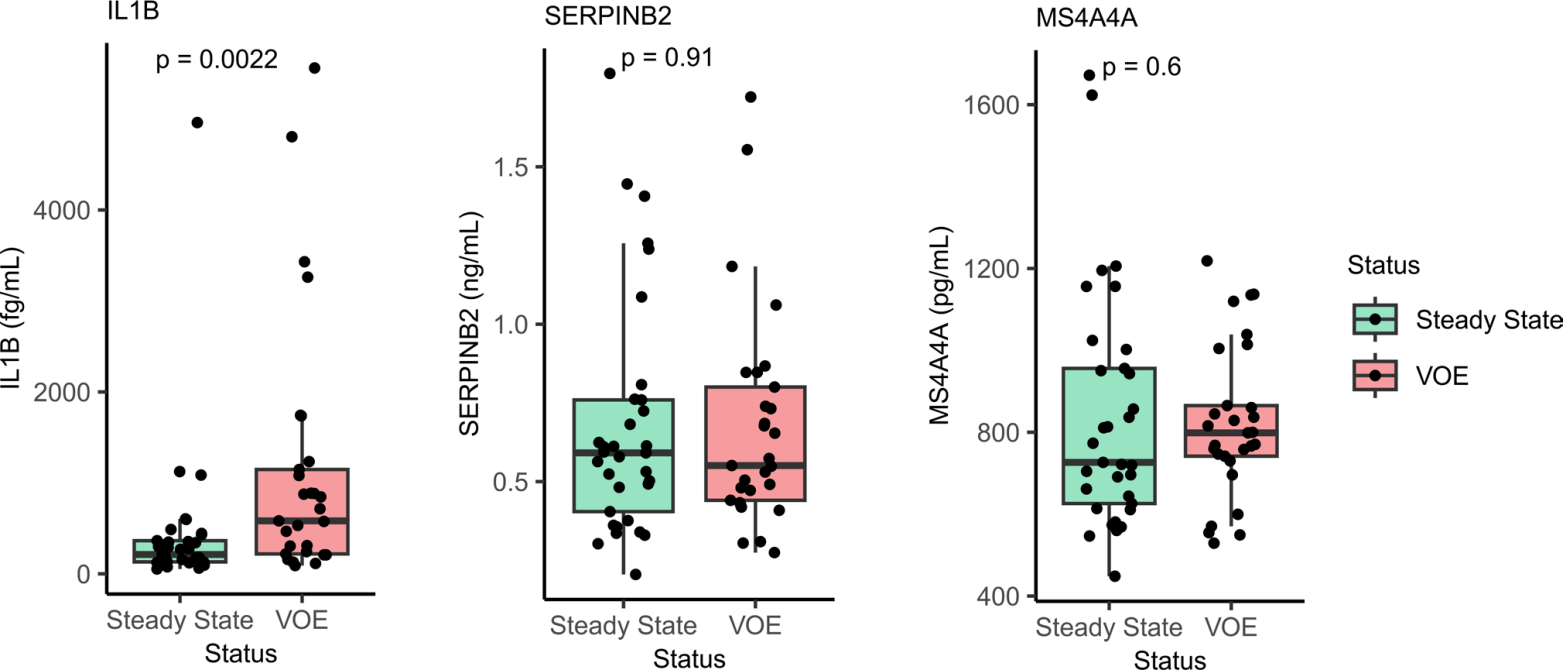
Protein expression of prospective biomarkers. Comparison of protein expression levels of 3 prospective biomarker genes between steady state and VOEs by Wilcoxon’s rank-sum test: IL-1B, MS4A4A, and SERPINB2, measured by S-PLEX and ELISA (*n* = 62 samples).

In addition to computational external validation, we measured the protein-level expression of these biomarkers in the plasma of 52 patients with SCD and compared their levels between steady state and VOEs (Figure 6). Among the 4 prospective biomarkers studied, IL-1B plasma protein levels were found to be significantly higher during VOEs when compared with steady state (*P* = 0.0022). MS4A4A and SERPINB2 protein levels were not found to be significantly different between steady state and VOEs in the plasma. We were not able to detect FAM20A protein expression in the collected plasma samples.

## Discussion

Although acute pain is a key clinical symptom of patients with SCD, and the most common reason for hospitalization, its impact on the transcriptome is still unclear. This gap in knowledge has impeded the development of diagnostic biomarkers for VOEs, which are essential for distinguishing between acute and chronic pain, and for establishing objective quantitative endpoints in clinical trials. Previously published studies have conducted transcriptome profiling of patients with SCD to describe gene expression changes between baseline and VOEs, but have been restricted by small sample sizes (13) or used microarrays for profiling (14, 15). In this study, we characterized the transcriptomic changes between steady state and VOEs in a dataset of more than 150 patients, including 19 patients for whom longitudinal data were available. If our results are substantiated in additional datasets, a feasible intervention may be to identify candidate diagnostic biomarkers for VOEs that can be developed for clinical care.

The analysis of the CD45^+^ cell–derived transcriptomes revealed that many of the genes upregulated during VOEs are associated with inflammatory processes. The cytokine transcripts *IL1A*, *IL1B*, and *IL10* were overexpressed during VOEs when compared with steady state; although the levels of these cytokines have been investigated extensively during pain episodes in SCD, the results have been inconsistent (16). The genes *CD177*, *FFAR3*, *SOCS3*, and *ANXA3* were also upregulated during VOEs, a finding that was previously reported in a whole-blood RNA-seq study of baseline and VOE transcriptional profiles (13). *CD177* is a marker of neutrophil activation, which further drives inflammation during VOEs (17). In addition, the levels of matrix metalloproteinase genes *MMP8* and *MMP9* were elevated during VOEs; these genes encode proteins that are released after neutrophil activation by proinflammatory mediators (18). Two genes selectively expressed in macrophages, *MS4A4A* and *SERPINB2*, were upregulated during VOEs when compared with steady state; macrophages are involved in extravascular hemolysis, which consequently promotes inflammation (19).

Acute pain episodes include changes in numerous immune mechanisms that are closely linked and involved in a multistep process, as illustrated by the results of the pathway enrichment analysis. The elevation of the heme metabolism pathway during VOEs can be attributed to the body’s response to increased hemolysis during an acute clinical complication (20). The release of free heme generates reactive oxygen species and increases oxidative phosphorylation, which can add to existing inflammation and exacerbate vaso-occlusion in a vicious cycle. The elevation of complement activation during VOEs is also typically triggered by hemolysis; the exact mechanism of complement activation is unclear, but early studies suggested activation via the alternative (complement-mediated) pathway (21). Similarly, upregulation of coagulation markers has also been linked to VOEs, since coagulation can lead to the activation of neutrophils and macrophages, resulting in thrombosis and organ damage (22, 23). The IL-6/JAK/STAT3 signaling pathway, often linked to chronic inflammation in cancer, was upregulated during VOEs when compared with steady state; the upregulation of this pathway has not previously been observed in the context of SCD (24). The elevation of the cholesterol homeostasis pathway during VOEs may be associated with hypocholesterolemia and altered lipid metabolism often observed in patients with SCD experiencing clinical complications (25, 26).

Patients with chronic pain often have hyperalgesia and are frequently hospitalized for VOEs (27). It is often clinically difficult to distinguish between VOE-related pain and daily pain by providers and patients alike. Our results demonstrate that patients with chronic pain exhibit differences in the CD45^+^ cell transcriptome during a VOE when compared with their baseline, which are similar to those observed in patients without chronic pain, but more extensive. All of the significant pathways identified during VOEs in the analysis of patients without chronic pain were also enriched during VOEs in patients with chronic pain, despite an overlap of only 18 significantly differentially expressed genes between the 2 subsets (GSEA utilizes the entire profile of transcripts, not just the significant ones). Notably, the IFN-γ response pathway was upregulated during VOEs; an elevation in IFN-γ levels has been previously connected to a frequent history of VOEs (28). Patients experiencing chronic pain exhibited increased elevation of the P13K/AKT/mTOR signaling pathway; this signaling cascade has been linked to neuropathic pain and opioid usage in multiple studies (29–32). We conclude that individuals with chronic pain who are hospitalized for VOEs typically exhibit even greater biological changes than in those patients without chronic pain who are hospitalized with VOEs, as well as additional expression changes. Further elucidation of these chronic pain–specific expression changes may add to our understanding of the sources of chronic pain, and lead to the development of biomarkers that may aid in identifying patients with chronic pain earlier.

The extent of transcriptomic changes was relatively low in CD71^+^ cells compared with CD45^+^, possibly because erythroid lineage cells at late stages of maturation to enucleated red cells have reduced their transcriptomic diversity. However, our results support a role for inflammation in SCD-linked pain in erythroid lineage cells, particularly in patients without chronic pain experiencing VOEs. The elevation of cell proliferation and oxidative phosphorylation pathways can be attributed to an increase in reticulocyte count that occurs due to hemolysis and destruction of erythrocytes during VOEs. If so, the differential expression is more likely associated with the patient’s response to the pain episode rather than driving the event. Similar to the CD45^+^ cells, in CD71^+^ cells, the IL-6/JAK/STAT3 signaling pathway, a major driver of inflammatory events, was upregulated during VOEs. The elevated expression of the IFN-α response pathway genes may be a consequence of increased hemolysis during a VOE (33). In a study of the proteome of neutrophils, the IFN-α signaling cascade was demonstrated to be upregulated in patients with SCD at steady state compared with healthy controls (34), and it is possible that neutrophil contamination may be influencing the CD71^+^ profiles.

Previous attempts at identifying biomarkers for VOE diagnosis relied on laboratory-based methods and often examined fewer than 10–15 candidate genes or proteins in small datasets without longitudinal samples. On the other hand, transcriptomic profiling enables the investigation of multiple candidate genes across larger sample sizes. Two of the 4 genes we identified as prospective biomarkers — *IL1B* and *MS4A4A* — have previously been associated with disease severity in SCD (14, 23). IL-1B is a proinflammatory cytokine (35), and MS4A4A regulates the expression of arginase in macrophages (36). SERPINB2 is a coagulation factor strongly expressed in activated macrophages. Little is known about the function of FAM20A in immune cells, but it may be involved in hematopoiesis (37). We attempted to validate the findings for 4 of the genes at the protein level, leading to confirmation of *IL1B* as a diagnostic biomarker for VOEs. The other 3 transcripts remain valid RNA markers but were not confirmed at the protein level for diverse reasons. MS4A4A is a membrane protein, and it is often challenging to quantify surface protein levels, particularly in plasma. We did not detect FAM20A in the collected plasma samples and thus were unable to validate its expression; this is likely because FAM20A is a Golgi-localized transmembrane protein, and current literature is ambiguous on whether it is secreted from expressing cells (37, 38). SERPINB2 expression may be required specifically in macrophages, whereas the plasma levels are contributed by multiple myeloid cell types (39, 40). Therefore, while still potentially relevant to the pathophysiology of acute pain events in SCD, additional studies using fresh cell collection would be needed to validate our transcriptomics findings. This technical challenge may make the plasma protein levels unsuitable as robust biomarkers. Further validation of IL-1B as a biomarker for acute pain events is warranted.

Our study has 3 major limitations. Firstly, the dataset comprised longitudinal samples from only 19 patients, which is insufficient to account for heterogeneity and hence address the likely predictive value of the potential biomarker genes for VOEs. Similarly, although we had collected follow-up samples after VOE resolution, we were not able to use these data to make comparisons between steady state versus follow-up, as well as VOE versus follow-up, since the number of follow-up samples was less than 20. However, we observed that the levels of gene expression for the 4 genes reduced during follow-up when compared with VOE in most of the follow-up samples. Future studies with larger numbers of longitudinal and follow-up samples will allow us to assess how gene expression varies across time points (such as determining whether there is a uniform return of gene expression levels after VOE to baseline), and to establish the sensitivity and reliability of the proposed diagnostic biomarkers. Both *IL1B* and *SERPINB2* are often expressed during injury or infection and hence may also be used for the diagnosis of other inflammatory conditions; while the upregulation of *IL1B* has been studied as a prospective biomarker for asthma (41) and inflammatory bowel disease (42), the elevation of *SERPINB2* has been linked to asthma (43) and leprosy (44). Thirdly, since most patients were on treatment with hydroxyurea (HU), we were not able to determine how the transcriptome of patients not on HU treatment may differ; we believe that the pathways elevated during VOEs are not associated with HU usage, since there was limited overlap of the pathways with our previous study examining the impact of HU treatment on the transcriptome (45).

To summarize, patients with SCD exhibit substantial changes in the transcriptome of immune cells and erythroid cells during VOEs when compared with steady state. VOE-associated gene expression is enriched for numerous biological mechanisms associated with inflammation, cell proliferation, and heme metabolism. Furthermore, patients with chronic pain displayed a 44% increased enrichment of statistically significant pathways during VOE when compared with patients without chronic pain. These findings, if validated in other cohorts and prospective, longitudinally followed patients, may provide valuable biomarkers to distinguish VOE from chronic pain. This, in turn, would influence strategies for the treatment of pain and facilitate the implementation of a multidisciplinary approach to chronic pain and earlier intervention. Mechanistic studies are needed to determine whether the observed changes are associated with VOEs or contributing to VOEs, to judge whether they are suitable as therapeutic targets to treat or prevent VOEs.

## Methods

### Sex as a biological variable.

Our study examined male and female participants, and sex was included as a covariate in the analyses.

### Study population.

The study population comprised 174 patients with sickle cell anemia, either HbSS or HbSβ^0^ thalassemia, receiving SCD care at Texas Children’s Hospital (TCH). All but 2 individuals in the cohort were at the maximum tolerated dose of HU during steady state, and 10 patients were on metformin treatment in addition to HU. A total of 215 CD45^+^ cell samples and 199 CD71^+^ cell samples were collected during steady state, VOEs, or a follow-up visit after the resolution of VOEs. Insufficient yield of RNA from CD71^+^ cells in some collections prevented analysis. For 19 patients, we collected samples during both steady state and VOEs, as well as during repeated VOE hospitalizations (Table 1). Chronic pain was assessed by chart review of opioid use frequency and clinical notes recorded during a patient’s routine visit detailing pain symptoms and duration. Patients experiencing chronic pain did not exhibit avascular necrosis or other comorbidities that would cause persistent pain. The proportion of patients in the cohort experiencing chronic pain was 8% to 10%, lower than reported at some institutions. This difference may be due to TCH prescribing practices designed to limit opioid use to the acute pain event period and avoid long-term opioid use that might contribute to opioid-induced hyperalgesia and chronic pain. Additional information on cohort demographics and longitudinal data is displayed in Supplemental Tables 7 and 8, respectively.

### Gene expression profiling.

A total of 3–5 mL of whole blood was collected in EDTA tubes for each patient at steady state at a scheduled outpatient clinic visit, during hospitalization for a VOE, or during a follow-up visit in clinic. The blood samples were incubated with Whole Blood CD45 Microbeads (Miltenyi Biotec), and CD45^+^ cells were extracted using an AutoMACS machine (Miltenyi Biotec). The CD45^–^ fraction was incubated with CD71 Microbeads (Miltenyi Biotec), and a similar separation was performed using the AutoMACS. RNA was collected from the CD45^+^ and CD71^+^ fractions using the Qiagen RNeasy kit. The quality and quantity of RNA was measured by UV spectroscopy. Globin depletion was performed on the CD71^+^ cell–derived RNA before sequencing (Illumina Globin Zero Gold kit). The quality of RNA was assessed using an Agilent 2100 Bioanalyzer with the Agilent RNA 6000 Nano Kit to generate the ratio of the 18S to 28S ribosomal subunits and to generate RNA integrity number (RIN) values greater than 7 for RNA-Seq analysis. The total RNA was normalized to 12.5 ng/μL. Poly-A selection and cDNA synthesis were performed with the TruSeq Stranded mRNA kit (Illumina). The libraries were sequenced on an Illumina NovaSeq 6000 instrument at the University of Washington Northwest Genomics Center to generate 2 × 101 bp paired-end reads.

We used FastQC (version 0.11.9) (46) to perform quality control of the sequencing libraries. Since the adapter contamination was minimal (<5%), the input reads were not further trimmed. The reads were aligned to the GRCh38 primary assembly and the GENCODE annotation (release 42) with STAR (version 2.7.10b) (47). The number of reads that mapped to each gene was calculated using HTSeq (version 2.0.2) (48) with the GENCODE annotation as reference. We removed genes with zero expression in at least 10% of the samples and normalized the raw read counts for library size with the trimmed mean of M values (TMM) method implemented in edgeR (49). Expression of the sex-linked genes *RPS4Y1* and *XIST* was used to verify the sex of each patient.

The normalized counts were subsequently log-transformed and provided as input to SNM (50) for the adjustment of age, sex, batch effect, pain status (chronic or no chronic pain), and metformin treatment status as fixed effect covariates. The time point was modeled as the biological variable of interest. Age was treated as a categorical variable, with samples being categorized as below 10 years, between 10 to 18 years, and above 18 years of age based on how they clustered during principal component analysis. Since CD45^+^ cells are primarily composed of T cells and B cells, we calculated the first principal component (PC1) scores of the 10 most representative genes for the T cell and B cell axes identified as blood informative transcripts (51); these scores were used to adjust for cell-type abundances with SNM. The adjusted gene expression matrix from SNM was provided as input for a paired DGE analysis with the limma-trend pipeline (52); the duplicateCorrelation function was used to model subject as a random effect. Genes with Benjamini-Hochberg–adjusted *P* values of less than 0.05 were considered to be differentially expressed between steady state and VOEs. While multiple VOE samples were collected for a few patients, analysis of these repeat profiles was not additionally informative.

The fgsea R package (11) with the Hallmark gene sets in the MSigDB (53, 54) database as the reference was used for determining enriched gene sets/biological pathways; the product of the negative logarithm of the adjusted *P* value multiplied by the log(fold change) of each gene was used as the ranking statistic. After ranking the genes, the enrichment score for each gene set was calculated by computing a running-sum statistic that indicates the degree of overrepresentation of a gene set in the ranked list. Pathways with an adjusted *P* value of less than 0.05 were considered significantly altered. To quantify whether there was a difference in pathway enrichment in patients without chronic pain compared with patients with chronic pain, we created 30 bootstrap subsets with equivalent sample sizes from all samples classified as no chronic pain and chronic pain and repeated the analyses. To compare pathway enrichment at the sample level, we computed the GSVA scores using the R package GSVA (55) with the SNM-transformed counts as input.

For the analysis of longitudinal data, we chose genes that were differentially expressed between steady state and VOEs and were involved in pathways significantly altered during VOEs. Subsequently, we plotted the changes in expression of these genes in patients with matched samples. To determine whether these genes could be proposed as potential biomarkers for VOEs, we calculated the PC1 scores of the genes for all individuals in the dataset. In addition, we validated these results in an external whole-blood transcriptomics dataset from a study by Creary et al. (13), adjusting for the abundance of T cells, B cells, and neutrophils.

### Protein expression analysis.

To examine the protein levels of the prospective VOE biomarkers, plasma samples were collected from 52 patients with sickle cell anemia; 33 samples were collected at steady state and 29 were collected during VOEs. The levels of IL-1B were measured by S-PLEX (S-PLEX Proinflammatory Panel 1, Mesoscale, K15396S-1), while MS4A4A, FAM20A, and SERPINB2 levels were measured by enzyme-linked immunosorbent assay (ELISA) (MS4A4A: Abbexa, abx531971; FAM20A: Thomas Scientific, CHM01D345; SERPINB2: Lifespan Biosciences, LS-F5568-1).

### Statistics.

The R package SNM was used to adjust for covariates. We identified differentially expressed genes with Limma (Benjamini-Hochberg *P* value < 0.05), and enriched pathways with fgsea. The calculation of the PC1 scores was performed using the stats package in R. Protein expression levels at steady state and VOEs were compared using the Wilcoxon rank-sum test (significance was defined as *P* < 0.05). In the box-and-whisker plots, the box shows the middle 50% of the data, and the upper and lower bounds of the boxes represent the first and third quartiles, respectively. The line in the middle denotes the median, and the whiskers extend to the minimum and maximum values. Outliers are represented with dots.

### Study approval.

Written informed consent was obtained from all patients prior to study participation. The study protocol was approved by the Baylor College of Medicine Internal Review Board.

### Data availability.

The bulk RNA-Seq data generated for this study are available in the NCBI GEO under accession number GSE291257. The R script for the DGE analysis is available on our GitHub repository: https://github.com/vtbhat/SCD_Omics_Scripts Values for all data points in graphs are reported in the Supporting Data Values file.

## Author contributions

VAS, AAP, and GKY conceptualized the study. VAS enrolled participants and collected data. APP curated the metadata for the study. VB performed data analysis and interpretation with supervision by GG. JJY and SP performed the protein-level validation. VB and VAS drafted the manuscript.

## Funding support

This work is the result of NIH funding, in whole or in part, and is subject to the NIH Public Access Policy. Through acceptance of this federal funding, the NIH has been given a right to make the work publicly available in PubMed Central.

- NIH grant R01HL164583.
- National Heart, Lung, and Blood Institute TOPMed Program/NIH grant R01HL164583.
- Pfizer.

## Supplementary Material

Supplemental data

Supplemental tables 1-8

Supporting data values

## Figures and Tables

**Table 1:**
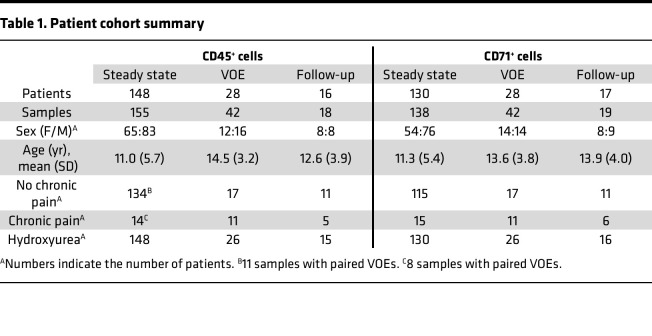
Patient cohort summary

## Version 1 03/09/2026

Electronic publication
