# Supplementary material for: Gene expression profiling identifies potential biomarkers for vaso-occlusive episodes in sickle cell disease
Source: JCI Insight. 2026 Mar 9;11(5):e193359. doi: 10.1172/jci.insight.193359 (PMC13041688; doi:10.1172/jci.insight.193359)
Supplement: Supplemental data [file jciinsight-11-193359-s043.pdf]

SUPPLEMENTAL TABLES

Table S1: List of genes differentially expressed between steady state and VOE in CD45+ cells in the complete cohort

Table S2: List of genes differentially expressed between steady state and VOE in CD45+ cells in patients without chronic pain

Table S3: List of genes differentially expressed between steady state and VOE in CD45+ cells in patients with chronic pain

Table S4: The gene expression levels of CD45, CD71, and CD15 for all CD71+ samples

Table S5: List of genes differentially expressed between steady state and VOE in CD71+ cells in the complete cohort

Table S6: List of genes differentially expressed between steady state and VOE in CD71+ cells in patients without chronic pain

Table S7: Complete summary of cohort demographics

Table S8: Per sample-level information for longitudinal samples

SUPPLEMENTAL FIGURES

Figure S1: Volcano plot depicting differentially expressed genes in CD45+ cells (n = 197 samples). The genes highlighted in brown are the prospective biomarker genes for VOE diagnosis

Figure S2: Heatmap showing the genes that most significantly contribute to the enrichment signal of the top five enriched pathways elevated during VOE when compared to steady state, ranked by adjusted p-values

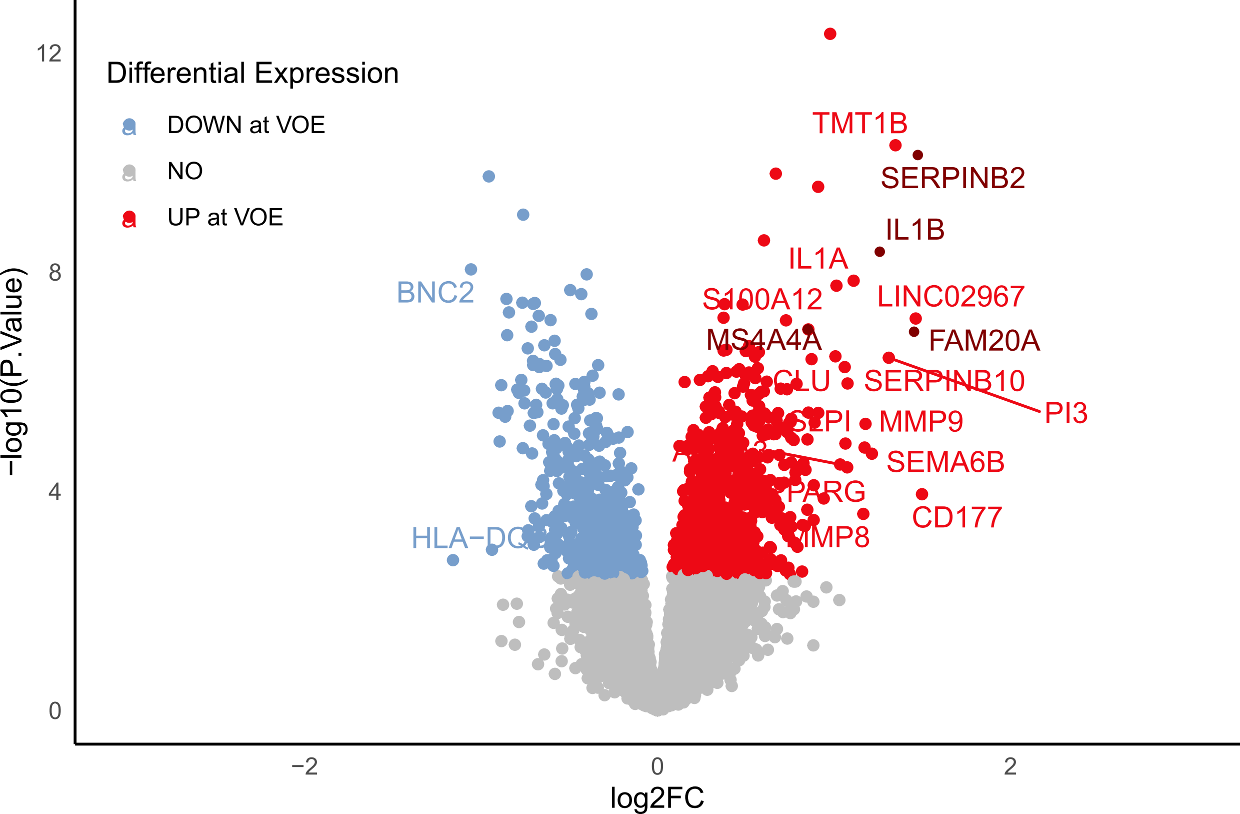

Differential Expression
DOWN at VOE
NO
UP at VOE
TMT1B
SERPINB2
IL1B
IL1A
S100A12
LINC02967
MS4A4A
FAM20A
CLU
SERPINB10
PI3
SLPI
MMP9
SEMA6B
PPARG
CD177
MMP8
BNC2
HLA−DQ
−log10(P.Value)
log2FC
12
8
4
0
−2
0
2

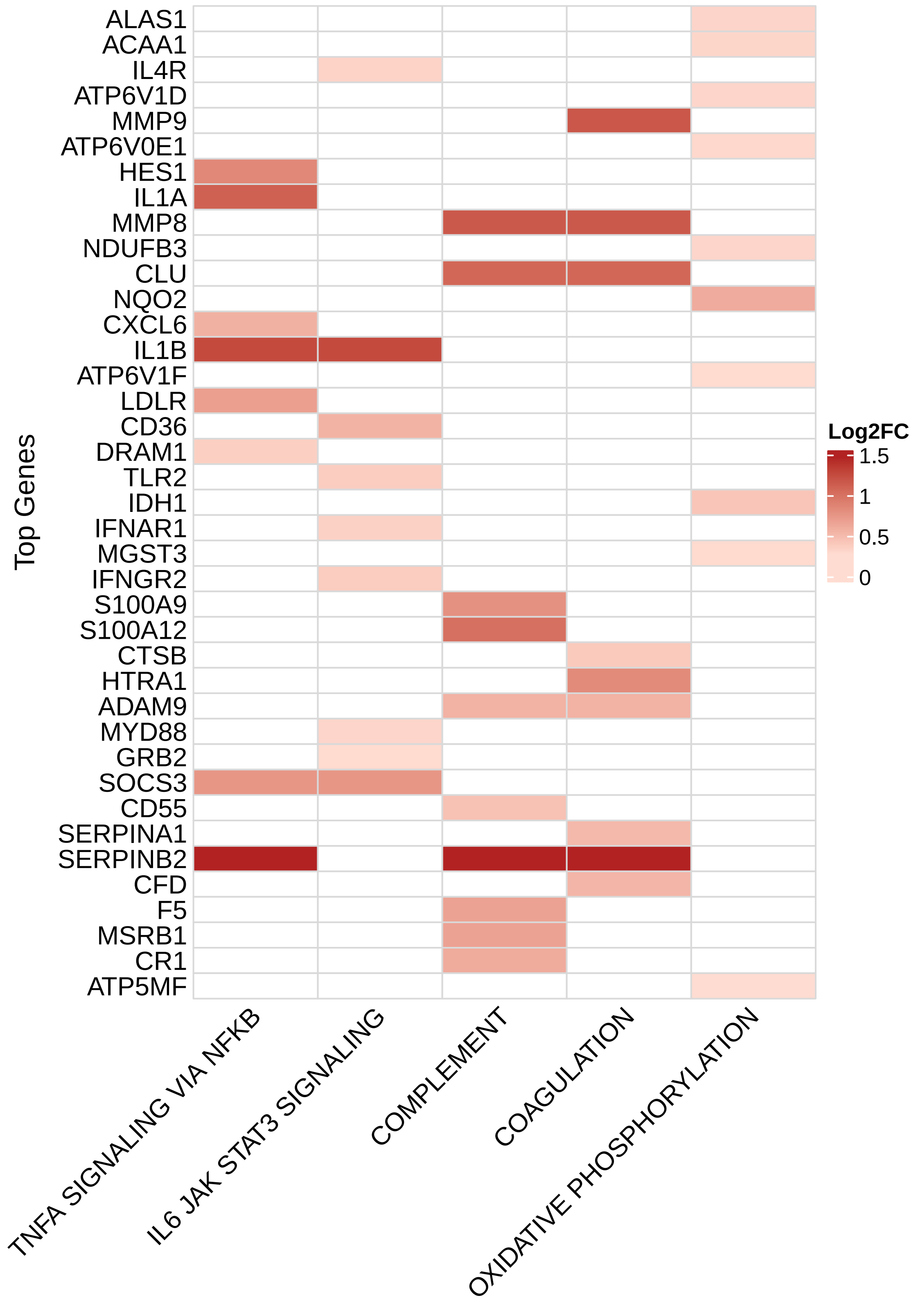

ALAS1
ACAA1
IL4R
ATP6V1D
MMP9
ATP6V0E1
HES1
IL1A
MMP8
NDUFB3
CLU
NQO2
CXCL6
IL1B
ATP6V1F
LDLR
CD36
DRAM1
TLR2
IDH1
IFNAR1
MGST3
IFNGR2
S100A9
S100A12
CTSB
HTRA1
ADAM9
MYD88
GRB2
SOCS3
CD55
SERPINA1
SERPINB2
CFD
F5
MSRB1
CR1
ATP5MF
Top Genes
TNFA SIGNALING VIA NFKB
IL6 JAK STAT3 SIGNALING
COMPLEMENT
COAGULATION
OXIDATIVE PHOSPHORYLATION
Log2FC
1.5
1
0.5
0
